# The Case for the Use of PPAR*γ* Agonists as an Adjunctive Therapy for Cerebral Malaria

**DOI:** 10.1155/2012/513865

**Published:** 2011-06-09

**Authors:** Lena Serghides

**Affiliations:** Sandra A. Rotman Laboratories, McLaughlin-Rotman Centre for Global Health, Toronto General Hospital, University Health Network, 101 College Street, Suite 10-359, Toronto, ON, Canada M5G 1L7

## Abstract

Cerebral malaria is a severe complication of *Plasmodium falciparum* infection associated with high mortality even when highly effective antiparasitic therapy is used. Adjunctive therapies that modify the pathophysiological processes caused by malaria are a possible way to improve outcome. This review focuses on the utility of PPAR*γ* agonists as an adjunctive therapy for the treatment of cerebral malaria. The current knowledge of PPAR*γ* agonist use in malaria is summarized. Findings from experimental CNS injury and disease models that demonstrate the potential for PPAR*γ* agonists as an adjunctive therapy for cerebral malaria are also discussed.

## 1. Introduction

Few diseases have the global health and economic impact of malaria [1]. In 2009, an estimated 225 million people were infected with malaria and close to a million people succumbed to their infection [2]. Malaria is caused by apicomplexan parasites belonging to the genus *Plasmodium*. Five species infect humans, *Plasmodium falciparum*, *P. vivax*, *P. ovale*, *P. malariae*, and most recently, *P. knowlesi* [3]. The majority of morbidity and mortality is caused by *P. falciparum* infection, with the highest burden born by children and pregnant women. In the absence of prompt and effective treatment, *P. falciparum* infection can progress quickly, rapidly becoming severe and fatal. The rise in drug-resistant parasites complicates the administration of effective treatment. 

Severe malaria has multiple manifestations that can occur singly or in combination. They include hyperparasitemia, high fever, haemoglobinuria, acute renal failure, acute pulmonary edema, metabolic acidosis and respiratory distress, hypoglycemia, anemia, and cerebral malaria, which is characterized by coma and convulsions. Cerebral malaria has the highest mortality rate of all the severe complications and is associated with long-term cognitive and neurological deficits in surviving children [4–6].

Intravenous artesunate is now the standard of care for severe malaria in both adults and children following the landmark SEAQUAMAT and AQUAMAT trials that demonstrated the superiority of artesunate over quinine in adults and in children [7, 8]. However, even with the improved efficacy of artesunate, fatality rates remained high, 15% in adults and 10.9% in children. Adjunctive therapies, defined as therapies administered in combination with antiparasitic drugs that modify pathophysiological processes caused by malaria, have been pursued as a way to improve the outcome of severe malaria. Adjunctive therapies may also help extend the efficacy of antiparasitic drugs, an important consideration given the emergence of artemisinin resistance [9, 10]. Several adjunctive therapeutic strategies have been tested in *P*. *falciparum* cerebral and severe malaria so far, unfortunately with-out much success (see [11] for a recent review). A number of adjunctive therapies (including nitric oxide, arginine, erythropoietin, levamisole) have demonstrated encouraging results in experimental models of cerebral malaria or in clinical trials in uncomplicated malaria and are awaiting evaluation in severe malaria [11].

This review will focus on the utility of PPAR*γ* agonists as an adjunctive therapy for the treatment of cerebral malaria. The current knowledge of PPAR*γ* agonist use in malaria will be summarized. We will also summarize data on additional mechanisms of action attributed to PPAR*γ* agonists that may be of benefit in cerebral malaria.

## 2. The Pathogenesis of Cerebral Malaria

Cerebral malaria is a severe complication of *P. falciparum* infection. It occurs in nonimmune individuals, with the greatest burden born by children in sub-Saharan Africa. Although the parasite is a key player in the development of cerebral malaria, hyperparasitemia does not necessarily correlate with disease severity, and cerebral pathology can develop even with the use of effective antiparasitic therapy. It has long been recognized that the host immune response plays an important role in mediating pathology in malaria, and this has fueled the search for effective immunomodulatory adjunctive therapies.

Sequestration of parasitized erythrocytes (PEs) in the microvasculature of the brain (and other organs), resulting in vascular occlusion and local tissue hypoxia and ischemia, is the hallmark feature of cerebral malaria [12]. Sequestration of PEs occurs via receptor-ligand interactions, with parasite-derived ligands expressed on the surface of PEs (a major one being *P. falciparum* erythrocyte membrane protein-1 or Pfemp-1) binding to receptors expressed on microvascular endothelial cells. Postmortem, in vitro, and genetic studies support that ICAM-1 is the major sequestration receptor for PEs in the brain, while the scavenger receptor CD36 is the major receptor outside the brain [13–21].

Parasites produce a variety of bioactive molecules that can elicit innate immune responses in the host [22]. An excessive inflammatory response with elevated levels of pro-inflammatory cytokines, especially TNF, is a major contributor to cerebral malaria pathology [23]. TNF, produced by activated endothelium and recruited leukocytes, can upregulate cell adhesion molecules, including ICAM-1, and exacerbate PE sequestration. Higher levels of TNF have been observed in cerebral malaria and correlated with mortality [24–26], and genetic predisposition to overproduce TNF in response to infection has been associated with susceptibility to cerebral malaria [27, 28]. Elevated levels of TNF are also seen in the cerebral spinal fluid (CSF) of infected children and correlated with encephalopathy [29]. Interestingly, CSF TNF levels did not correlate with serum levels, implying independent cerebral generation of TNF. Elevated levels of additional inflammatory mediators including IFN*γ*, IL-6, IL-1*β*, IL-1ra, IL-10, MIP-1*α* and MIP-1*β*, MCP-1, and IP-10 have been observed in cerebral malaria patients [26, 30–34].

Parasite sequestration and inflammation can lead to endothelial activation and dysfunction. Activated endothelium can lead to monocyte and platelet recruitment further impeding vessel flow and contributing to tissue hypoxia and ischemia [35]. Widespread endothelial activation (including increased ICAM-1 expression and the disruption of cell-junction proteins) has been observed in postmortem studies of cerebral malaria patients [36], and markers of endothelial activation and dysfunction such as soluble ICAM-1, von Willebrand factor, and angiopoietin-2 are elevated in cerebral malaria [37–39]. Low nitric oxide (NO) bioavailability (potentially due to quenching by cell-free hemoglobin released during hemolysis) contributes to the development of endothelial dysfunction in malaria infection [40, 41]. 

Sequestration, inflammation, and endothelial dysfunction can lead to a breakdown of the blood-brain barrier (BBB). Hemorrhages are common autopsy findings in cerebral malaria [12, 42, 43], as are focal disruptions of the BBB [44]. The activation of perivascular macrophages and axonal damage observed in cerebral malaria may be the result of cytokines, parasite antigens, and plasma proteins crossing the BBB, in addition to local hypoxic and inflammatory conditions [36, 45, 46]. 

Metabolic perturbations are also common in children with cerebral malaria and may contribute to pathology. Vascular obstruction leading to hypoxia, or TNF-induced cytopathic hypoxia, have been proposed as possible causes [47–49].

Recent investigations using fluorescein angiography and fundoscopy have permitted a view of the brain microvasculature in living patients with cerebral malaria, by imaging the retina (the only part of the central nervous system (CNS) vasculature that is available for direct observation). Pediatric cerebral malaria patients had evidence of PE sequestration and thrombi (containing both fibrin and platelets) in their vasculature that were associated with perfusion abnormalities and areas of ischemia and tissue damage (retinal whitening). Focal disruptions of the BBB were observed most often, but not always, in association with hemorrhages [44, 50, 51]. Postmortem analysis revealed axonal damage not only in areas of hemorrhage but also in areas of vascular occlusion by sequestered parasites and/or fibrin-platelet thrombi [51].

Cerebral malaria is a complex disorder that is as yet not fully understood. Multiple processes likely contribute to its development including peripheral and CNS inflammation, PE sequestration, vascular endothelial activation, prothrombotic activation, blood flow obstruction, tissue hypoxia and ischemia, metabolic changes, and BBB dysfunction, leading to neurodegeneration. These processes can contribute to the seizures and coma seen in cerebral malaria patients and the neurologic and cognitive deficits which persist in a portion of cerebral malaria survivors [4, 29]. The activation of PPAR*γ* appears to play an important role in recovery in several models of CNS injury and disease, by limiting inflammation and cytotoxicity and promoting reparative mechanisms. These mechanisms may also be protective in the context of cerebral malaria as well. Interestingly, PPAR*γ* was one of only two genes in a malaria-resistance locus identified using a genome-wide analysis of inbred mouse lines [52], supporting a protective role for PPAR*γ* in malaria.

## 3. PPAR*γ* and Its Agonists

PPAR*γ* is a member of the family of nuclear hormone receptors which function as ligand-activated transcription factors [53]. PPAR*γ* endogenous ligands include oxidized fatty acids and prostanoids, and synthetic ligands include the thiazolidinedione (TZD) class of antidiabetic drugs (e.g., rosiglitazone and pioglitazone). Upon ligand activation, PPAR*γ* heterodimerizes with the retinoid X receptor (RXR), a nuclear receptor for 9-cis-retinoic acid. The ligand-bound PPAR*γ*-RXR heterodimer regulates gene transcription by binding to conserved DNA sequences called PPRE (PPAR response elements) on target genes. PPAR*γ* can also regulate other transcription factors, through nongenomic trans-repression, where the inhibition of transcription occurs by preventing the dissociation of corepressors or by sequestering the coactivators necessary for the binding of the transcription factor to DNA [54].

Originally characterized in adipocytes as a regulator of lipid and glucose metabolism, current evidence indicates that PPAR*γ* is present in most cell types (including immune cells, endothelial cells, and neurons) and mediates multiple functions in both physiological and pathological conditions [55, 56]. 

PPAR*γ* agonists have been extensively studied in many inflammatory settings, in vitro, in animal models, and in humans, and in most cases they have demonstrated anti-inflammatory properties [57]. These anti-inflammatory properties are early events (observed prior to any metabolic effects) and occur even with low-dose administration of the agonists [58]. PPAR*γ* agonists can inhibit proinflammatory responses from a variety of cells including macrophages, dendritic cells, T cells, endothelial cells, vascular smooth muscle cells, microglia, and astrocytes [59–74]. The anti-inflammatory properties of the agonists are mediated by the transrepression effects of activated PPAR*γ* on transcription factors including activator protein-1 (AP-1), signal transducers and activators of transcription 1 (STAT-1), nuclear factor kB (NF-*κ*B), and nuclear factor of activated T cells (NFAT). PPAR*γ* agonists can also suppress inflammation by PPAR*γ*-independent mechanisms, for example, the suppression of JAK-STAT-dependent inflammatory responses in activated microglia and astrocytes via the induction of members of the suppressor of cytokine signaling (SOCS) family [75, 76]. 

Data have also been accruing on the neuroprotective properties of PPAR*γ* agonists in models of CNS injury, ischemic stroke, and diseases of the CNS including multiple sclerosis, ALS, and Parkinson's disease [77–80]. These data suggest that PPAR*γ* may be involved in coordinating cellular responses to CNS injury and disease. The potential benefit of the anti-inflammatory and neuroprotective properties of PPAR*γ* agonists in cerebral malaria will be discussed below.

## 4. Generation of Endogenous PPAR*γ* Ligands in Malaria Infection

*Plasmodium falciparum* may itself activate PPAR*γ*, perhaps as part of a strategy aimed at enhancing symbiotic survival between the parasite and the host. Hemozoin, a pigment produced by *Plasmodium* to detoxify free heme generated by the degradation of haemoglobin [81], can produce large amounts of hydroxyl-fatty acids, including 15-hydroxyecosatetraenoic acid (15-HETE), 13-hydroxyoctadecadienoic acid (13-HODE), and 4-hydroxynonenal (4-HNE) by heme-catalyzed lipoperoxidation [82]. 15-HETE and 13-HODE are specific ligands of PPAR*γ*, and 4-HNE is an inducer of PPAR*γ* [83]. Hemozoin-mediated immunosuppressive effects on myeloid cell functions including phagocytosis, inflammatory responses, oxidative burst, and dendritic cell differentiation and maturation have been reported [84–89]. Hemozoin was able to induce the upregulation of PPAR*γ* mRNA, while the inhibition of PPAR*γ* reversed some of the hemozoin-mediated effects, suggesting that the immunomodulatory effects of hemozoin may be, at least partly, mediated by PPAR*γ* activation [90].

## 5. The Use of PPAR*γ* Agonists in Malaria: What We Know So Far

The use of PPAR*γ* agonists to modulate immune responses to malaria was initially motivated by reports demonstrating that PPAR*γ* regulates CD36 transcription and that PPAR*γ* agonists have anti-inflammatory properties [61, 63]. 

At that time, the scavenger receptor CD36 was revealed to be a major, noninflammatory, phagocytic receptor for nonopsonised mature-stage PEs [91]. It was speculated that CD36-mediated phagocytosis of PEs represented an innate immune mechanism for controlling the parasite burden in nonimmune individuals (who are most at risk of developing severe disease) [91–94]. Later, CD36-mediated phagocytosis of ring-stage PEs and stage I and IIa gametocytes was also reported [95, 96]. The importance of CD36-mediated innate control of acute blood-stage malaria was demonstrated in vivo, in a murine model of hyperparasitemia (*P. chabaudi* AS infection) [97]. In this model, mice deficient in CD36 had higher parasitemia levels and higher mortality compared to CD36-sufficient mice [97]. 

Various PPAR*γ* agonists including the natural ligands 15d-PGJ2 and 9-cis-retinoic acid (which binds RXR to activate the PPAR*γ*-RXR heterodimer), and the synthetic TZDs, ciglitazone, troglitazone, and rosiglitazone, were shown to upregulate the CD36 expression on monocytes and enhance the CD36-mediated phagocytosis of PEs [92, 95, 96, 98]. And unlike Fc-mediated phagocytosis, CD36-mediated uptake of PEs occurred in a noninflammatory manner that was not associated with release of TNF or IL-6 [91, 99]. This process appeared similar to the CD36-mediated clearance of apoptotic cells, which is also non-inflammatory, but did not appear to involve cooperation with integrins [91, 100, 101]. PPAR*γ* agonists also dramatically upregulated the uptake of ring-stage PEs and gametocytes [95, 96]. These findings where extended in vivo using the mouse model of hyperparasitemia. Mice receiving rosiglitazone had lower parasitemia compared to controls [102]. This reduction in parasitemia was CD36 dependent, as it was not observed in mice deficient in CD36. 

These data are consistent with the reported ability of PPAR*γ* activation to polarize macrophages towards an alternatively activated phenotype [57]. Alternatively activated macrophages have reduced expression of proinflammatory cytokines, enhanced expression of anti-inflammatory cytokines, in particular IL-10, and enhanced expression of pattern-recognition receptors, including CD36. They have been implicated in pathogen sequestration, wound healing, and phagocytosis of apoptotic cells. In the context of malaria, alternatively activated macrophages could help control parasite burden while limiting associated inflammation, thus reducing host pathology [103]. 

Although reducing the parasite burden by enhancing phagocytic clearance of parasites (especially ring-stage PEs) will undoubtedly be beneficial to the outcome of infection and may be a contributing mechanism to the genetic resistance offered by hemoglobinopathies (sickle cell and both *α*-thalassemia and *β*-thalassemia) and glucose-6-phosphate dehydrogenase and pyruvate kinase deficiencies [104–106], in the context of cerebral malaria, parasitemia levels are not correlated with disease severity. Rather, inflammation, and especially TNF levels seem to correlate with disease severity, encephalopathy, and death [23, 29]. Thus, the anti-inflammatory properties of PPAR*γ* agonists may be their most important quality when it comes to the treatment of cerebral malaria.

Human monocytes and murine macrophages treated with PPAR*γ* agonists generate significantly less TNF in response to malaria-related inflammatory stimuli including parasite lysates and *P. falciparum* glycosylphosphatidyl inositol (GPI), a malaria toxin that interacts with TLR2 [98, 107, 108]. This was associated with the inhibition of NF-*κ*B and MAPK signaling [102]. PPAR*γ* is known to inhibit the NF-*κ*B signaling [54], and a PPAR*γ*-mediated inhibitory effect on MAPK signaling has recently been described [109]. However, whether the anti-inflammatory effects of the agonists were related to PPAR*γ* activation was not directly examined. 

The effects of PPAR*γ* agonists in vivo have been tested in a mouse model of experimental cerebral malaria (*P. berghei* ANKA). Cerebral pathology in this model is the result of an uncontrolled proinflammatory response to infection [47, 110]. Infected mice treated with rosiglitazone had a more balanced inflammatory response, with reduced plasma levels of TNF, a reduced TNF to TGF*β* ratio, and higher IL-10 levels ([102], and unpublished results by Serghides et al.). Mice receiving rosiglitazone were also protected from developing signs of cerebral pathology and had significantly improved survival rates. This was evident even when rosiglitazone was administered as late as 5 days postinfection, just prior to the initiation of cerebral pathology [102]. The effects of rosiglitazone treatment on endothelial dysfunction and cerebral pathology in this model are currently under investigation in our lab. 

Given the encouraging data in the mouse models, a phase I/IIa randomized double-blind placebo-controlled trial was undertaken to test the safety, tolerability, and efficacy of rosiglitazone adjunctive therapy in 140 Thai adults with uncomplicated *falciparum* malaria [111]. Rosiglitazone (4mg twice daily for 4 days) was administered as an adjunctive therapy in combination with atovaquone-proguanil and was found to be safe and well tolerated. Patients receiving rosiglitazone had significantly reduced 50% and 90% parasite clearance times, with the mean 90% parasite clearance time being reduced by 25% in the rosiglitazone group (from 40.4 h in placebo to 30.9 h in the rosiglitazone group). It is tempting to speculate that improved parasite clearance was due to enhanced CD36-mediated clearance, but direct evidence is lacking. However, these findings do corroborate the effects of rosiglitazone on parasitemia observed in the mouse model, a process that was CD36 dependent [102]. A nonstatistically significant trend towards greater fever clearance at 4 hours posttreatment was observed in those receiving rosiglitazone (43% afebrile in the rosiglitazone group compared to 27% afebrile in the placebo group, *P* = .073). Patients receiving rosiglitazone also had significantly lower levels of IL-6 and MCP-1 and trended towards significantly lower levels of TNF at 24 and 48 hours posttreatment [111]. Both the fever reduction and the lower levels of proinflammatory biomarkers suggest that treatment with rosiglitazone was associated with anti-inflammatory effects that were obvious early during the course of therapy in these patients.

The findings in the rosiglitazone trial share some similarities to those of a randomized trial of vitamin A supplementation in children from Papua New Guinea [112]. 9-cis-retinoic acid is a metabolite of vitamin A and an agonist of PPAR*γ* (via RXR ligation), and like rosiglitazone, has been shown to enhance CD36-mediated PE uptake and reduce malaria-induced TNF production in vitro [113]. Children supplemented with vitamin A had lower parasitemia levels and fewer febrile episodes than did children in the control group, although both groups had the same rate of infection [112], suggesting a common mechanism of enhanced innate clearance of PEs and reduced inflammation.

## 6. Lessons from the Use of PPAR*γ* Agonists in Neuroinflammatory and Neurodegenerative Diseases

Data on the anti-inflammatory and neuroprotective properties of PPAR*γ* agonists in models of neuroinflammatory and neurodegenerative disease states may give us an insight into how PPAR*γ* agonist could function in cerebral malaria [77–80].

Relevant to cerebral malaria pathology, PPAR*γ* is expressed not only in immune cells and in peripheral organs, but also in the CNS (microglia, astrocytes, perivascular macrophages, oligodendrocytes, and neurons) and in human brain microvascular endothelial cells [114–116]. Further, PPAR*γ* agonists such as rosiglitazone and pioglitazone can cross the BBB [117], and thus, can exert their effects not only peripherally but also directly on the CNS. 

As mentioned above cerebral malaria is an inflammatory disease [23, 49]. Proinflammatory cytokines, especially TNF, initiate an inflammatory cascade that leads to endothelial activation, cell adhesion molecule upregulation, enhanced PE, leukocyte- and platelet-endothelial adhesion, endothelial dysfunction, and BBB breakdown [47]. Perivascular macrophages, astrocytes, and microglia are also activated in cerebral malaria and can produce inflammatory mediators leading to neuronal damage [118]. Several anti-inflammatory properties of relevance to cerebral malaria pathology have been ascribed to PPAR*γ* agonists. PPAR*γ* agonists have been shown to inhibit the following: the expression of inflammatory mediators, such as TNF, IL-6, IL-1b, and COX-2, from activated monocytes and microglial [74, 119]; the release of chemokines including MCP-1, MIP-1a, and MIP-1b; the expression of chemokine receptors on leukocytes; the inflammation-induced upregulation of cell adhesion molecules on vascular endothelium, including ICAM-1 [120, 121]; the recruitment of leukocytes to injured sites [74, 122]; the release of matrix metalloproteinases (which degrade the extracellular matrix and contribute to BBB dysfunction) from macrophages and glial cells [123, 124]. In the context of cerebral malaria, these activities could result in less proinflammatory cytokines peripherally and in the CNS, a reduction in PE adhesion and leukocyte recruitment in the brain, and protection of the BBB integrity. 

Malaria is associated not only with inflammation, but also with oxidative stress, conditions that together can lead to increased cytotoxicity. Elevated levels of TNF in addition to oxidants such as superoxide and free heme can lead to neuronal damage [125, 126]. TNF, superoxide, and free heme (caused by hemolysis) are all elevated in cerebral malaria and may contribute to the neuronal damage detected in brains of cerebral malaria patients [43, 45, 46, 127]. In addition to their anti-inflammatory properties, PPAR*γ* agonists also have antioxidant properties. PPAR*γ* agonists enhance the endothelial and neuronal expression and activity of superoxide dismutase-1 (SOD-1) and catalase (both of them have functional PPREs in their promoter) [128–132]. SOD-1 and catalase detoxify superoxide by catalyzing its conversion into water and oxygen. PPAR*γ* can also suppress superoxide generation by decreasing the expression of components of the NAD(P)H oxidase complex [129, 130, 133]. Rosiglitazone-induced reduction in NAD(P)H oxidase activity has been detected in models of hypertension and diabetes [128, 134]. Heme oxygenase-1 (HO-1) also contains a PPRE in its promoter and can be upregulated by PPAR*γ* activation [135]. HO-1 is induced during conditions of oxidative stress and catalyses the breakdown of heme into biliverdin, iron, and CO. CO is anti-inflammatory and can inhibit TNF while inducing IL-10 release [136]. HO-1 induction protects astrocytes from heme-mediated oxidative injury, and astrocytes deficient in HO-1 are much more susceptible to cell death [137]. HO-1 and CO have been shown to be protective in experimental cerebral malaria and were associated with reduced inflammation, protection of the BBB, and enhanced survival [138]. 

Oxidative stress can also result in decreased NO bioavailability, via scavenging by cell-free hemoglobin and/or superoxide-mediated formation of the toxic peroxynitrite [139]. Low NO bioavailability has been associated with disease severity, while NO supplementation improves disease outcome in human and experimental cerebral malaria ([40, 41, 140–143], submitted by Serghides et al.). By enhancing cell-free hemoglobin detoxification (via HO-1 upregulation) and by reducing the levels of reactive oxygen species (via SOD-1 and catalase upregulation), PPAR*γ* agonist activity may enhance NO bioavailability [144]. A trial in diabetic patients is currently underway examining whether pioglitazone will improve NO bioavailability (clinicaltrials.gov ID NCT00770367).

An additional neuroprotective property of PPAR*γ* agonists is their ability to regulate the expression of the glutamate receptor GLT1/EAAT2 (GLT1/EAAT2 has six putative PPREs in its promoter region) [145]. Glutamate is the major excitatory neurotransmitter in the mammalian CNS, but high amounts of glutamate released in the intersynaptic spaces can cause neurodegeneration and excitotoxic neuronal death. Glutamate plays an important role in many CNS pathologic conditions including ischemia, trauma, and neurodegenerative disorders [146]. Glutamate levels have not been measured in humans but were shown to be elevated in the CSF and in the cerebral cortex of mice with experimental cerebral malaria, suggesting that glutamate toxicity may occur in cerebral malaria. In these mice, glutamate levels correlated with the development of cerebral symptoms [147, 148]. The mechanism for maintaining low extracellular glutamate levels is astrocytic uptake via glutamate transporters including GLT1/EAAT2, which is responsible for the removal of up to 90% of extracellular glutamate. PPAR*γ* agonists increased astrocytic expression of GLT1/EAAT2 mRNA and protein in vitro [145] and protected astrocytes and neurons from glutamate-induced cell death [145, 149, 150]. In rats, rosiglitazone prevented the stress-induced decrease in synaptosomal glutamate uptake, by enhancing glial expression of GLT1/EAAT2 [151].

Collectively these data support a neuroprotective role for PPAR*γ* agonists via the attenuation of inflammation, oxidative stress, and cytotoxicity [152]. Such protective effects have been observed with PPAR*γ* agonist use in models of ischemic and hemorrhagic stroke [153–158], and in models of CNS disease including Alzheimer's disease, multiple sclerosis (MS), amyotrophic lateral sclerosis, and Parkinson's disease [152]. In the ischemic models, PPAR*γ* agonist use was associated with reduced brain injury and with improved neurological outcomes [124, 154, 159–162]. In the CNS disease models, PPAR*γ* agonists attenuated neuron loss, prevented motor dysfunction, improved motor performance, and reversed memory decline [163–166]. Supporting data from human trials also exist. In a pilot study in Alzheimer's patients, rosiglitazone administration improved cognitive function [167, 168]. In a small placebo-controlled trial of pioglitazone use in patients with relapsing MS, gray matter atrophy and lesion burden, as assessed by MRI, were reduced in the pioglitazone group [169]. Diabetic patients receiving pioglitazone or rosiglitazone had improved functional recovery after stroke compared to patients not taking TZDs [170]. Clinical trials are underway testing the efficacy of TZDs in Alzheimer's (phase III), ALS (phase I/II), and Friedreich's ataxia (pilot).

## 7. Are PPAR*γ* Agonists Promising Candidates for Adjunctive Therapy in Cerebral Malaria?

PPAR*γ* activation may enhance the tolerance of the host to malaria infection by immunoregulatory mechanisms (modulation of the inflammatory response to infection), and by mechanisms that render tissues more resistant to inflammatory damage. Such immunomodulatory effects are likely to be protective in the context of cerebral malaria. However, whether PPAR*γ* activation following the onset of cerebral malaria (once the inflammatory cascade has begun) will be protective is an open question. Other immunomodulatory therapies tested in cerebral malaria in the past (e.g., anti-TNF antibodies, dexamethasone) have failed [11]. That PPAR*γ* activation impacts several pathways and may have not only neuroprotective but also neuroregenerative effects improves the likelihood of efficacy. However, it is unknown whether the regenerative effects seen with long-term PPAR*γ* agonist use in chronic CNS disease will also be obvious with a short treatment course, as would be administered in cerebral malaria.

Rosiglitazone (4 mg twice daily for 4 days administered in combination with atovaquone-proguanil) was found to be safe and well tolerated in uncomplicated malaria. Mean serum glucose, alanine aminotransferase, and aspartate aminotransferase levels did not differ between patients receiving placebo and those receiving rosiglitazone [111]. In addition, there were no differences observed in the incidences of adverse events including headache, myalgia, weakness, nausea, vomiting, diarrhea, or palpitations between the two groups [111]. TZDs are antidiabetic drugs, and so a concern would be the possible exacerbation of the hypoglycemia commonly seen in severe malaria; however, rosiglitazone and other TZDs function as insulin sensitizers and are generally not known to cause hypoglycemia, and as mentioned above, rosiglitazone did not cause hypoglycemia in patients with uncomplicated malaria [111]. Rosiglitazone may also worsen edema by increasing fluid retention, but clinically significant fluid retention tends to occur only with long-term use [171]. Increased risk of myocardial infarction and hepatotoxicity are risk factors associated with rosiglitazone use, but again these are complications associated with long-term use [172]. Finally, it is worth considering whether PPAR*γ* agonists could have an impact on the acquisition of adaptive immunity to malaria via modulatory effects on dendritic cells, T cells, and B cells [57]. 

The existing data on the use of PPAR*γ* agonists in malaria are encouraging, with rosiglitazone being safe, well tolerated, and efficacious in uncomplicated malaria patients. Given the anti-inflammatory, neuroprotective, and neuroregenerative properties reported for PPAR*γ* agonists in models of CNS injury, ischemic stroke, and diseases of the CNS, we can hypothesize that PPAR*γ* activation in cerebral malaria may lead to improved outcome and possibly less long-term cognitive and neurological deficits. However, a randomized double-blind placebo-controlled trial in patients with cerebral malaria will be required to determine if these hypotheses are correct.
